# Triazoloacridone C-1305 impairs *XBP1* splicing by acting as a potential IRE1α endoribonuclease inhibitor

**DOI:** 10.1186/s11658-021-00255-y

**Published:** 2021-03-17

**Authors:** Sylwia Bartoszewska, Jarosław Króliczewski, David K. Crossman, Aneta Pogorzelska, Maciej Bagiński, James F. Collawn, Rafal Bartoszewski

**Affiliations:** 1 Department of Inorganic Chemistry, Medical University of Gdansk, Hallera 107, 80-416 Gdańsk, Poland; 2 Department of Biology and Pharmaceutical Botany, Medical University of Gdansk, Hallera 107, 80-416 Gdańsk, Poland; 3 Department of Genetics, Heflin Center for Genomic Science, University of Alabama at Birmingham, Birmingham, AL 35233 USA; 4 Department of Organic Chemistry, Medical University of Gdansk, Gdansk, Poland; 5 Department of Pharmaceutical Technology and Biochemistry, Faculty of Chemistry, Gdansk University of Technology, 80-233 Gdańsk, Poland; 6 Department of Cell, Developmental and Integrative Biology, University of Alabama at Birmingham, Birmingham, AL 35233 USA

**Keywords:** XBP1s, UPR, ER stress, IRE1α

## Abstract

Inositol requiring enzyme 1 alpha (IRE1α) is one of three signaling sensors in the unfolding protein response (UPR) that alleviates endoplasmic reticulum (ER) stress in cells and functions to promote cell survival. During conditions of irrevocable stress, proapoptotic gene expression is induced to promote cell death. One of the three signaling stressors, IRE1α is an serine/threonine-protein kinase/endoribonuclease (RNase) that promotes nonconventional splicing of *XBP1* mRNA that is translated to spliced XBP1 (XBP1s), an active prosurvival transcription factor. Interestingly, elevated IRE1α and XBP1s are both associated with poor cancer survival and drug resistance. In this study, we used next-generation sequencing analyses to demonstrate that triazoloacridone C-1305, a microtubule stabilizing agent that also has topoisomerase II inhibitory activity, dramatically decreases *XBP1s* mRNA levels and protein production during ER stress conditions, suggesting that C-1305 does this by decreasing IRE1α’s endonuclease activity.

**Supplementary Information:**

The online version contains supplementary material available at 10.1186/s11658-021-00255-y.

## Introduction

During tumor development and progression, transformed cells adapt to increased demands on protein and lipid production that are needed for rapid proliferation [1] by enhancing endoplasmic reticulum (ER) function and expansion. To accomplish this, cancer cells take advantage of the adaptive multifunctional signaling pathway called the unfolded protein response (UPR) [1]. The normal function of this pathway is to protect cells against the accumulation of unfolded or misfolded proteins in ER. The UPR does this by activating three ER transmembrane sensors: inositol-requiring protein 1 alpha (IRE1α encoded by *ERN1*), protein kinase RNA-like ER kinase (PERK) and activating transcription factor 6 (ATF6) [2]. The function of the UPR is to promote cell survival and restore proper ER function, or alternatively during irrevocable stress, to trigger cell death when cellular homeostasis cannot be restored [3]. Interestingly, cancer cells avoid this UPR transition from prosurvival to apoptosis, and therefore strategies that inhibit the prosurvival pathways have become an attractive target for novel anticancer therapies.

Although the all three UPR sensors provide appealing therapeutic candidates, IRE1α activity has been a major focus because it promotes a protumoral phenotype in several cancers and furthermore, elevated levels of IRE1α are associated with poor cancer prognosis (reviewed in [4, 5]). IRE1α is ubiquitously expressed and displays both serine/threonine kinase and endoribonuclease (RNase) activities. Activation of IRE1α’s RNAse activity requires trans-auto phosphorylation and subsequent oligomerization of IRE1α [6]. Once activated, IRE1α’s RNAse is utilized to degrade ER bound RNAs, termed as regulated IRE1α-dependent decay (RIDD). A second important function is to remove a 26-base intron from X-box binding protein 1 (*XBP1)* mRNA in an unconventional splicing reaction that produces a highly active spliced XBP1 (XBP1s) transcription factor [7–12].

XBP1s’s function is to enhance the expression of ER-resident chaperones and genes involved in ER associated protein degradation (ERAD) [13] as well as ER expansion [11].

Given that dysregulation of IREα and XBP1s levels are associated with poor cancer patient survival and drug resistance [14–19] and IRE1’s kinase and endoribonuclease activities are required for generating XBP1s, IRE1α has become an attractive therapeutic targets for novel anticancer therapies. Although numerous small molecules that inhibit IRE1α activity has been developed, their use in cancer therapy remains limited [4, 5], and therefore the search for new drug candidates that inhibit IRE1α activity is clearly needed. In this study, we demonstrate that triazoloacridone C-1305, a microtubule stabilizing agent that also has topoisomerase II inhibitory activity [20, 21], efficiently prevents XBP1 splicing, therefore suggesting that C-1305 inhibits IRE1’s endoribonuclease activity. Given C-1305’s multiple activities, it could be considered as a potential lead compound to include in the development of future cancer therapeutic strategies.

## Materials and methods

### Cell lines and culture conditions

Human bronchial epithelial 16HBE14o-cells were obtained as previously described (Sigma-Aldrich, Catalog no. #SCC150) [22]. HeLa S3 cells (ATCC CCL‐2.2) and human non-small lung carcinoma A549 cells (ATCC CCL-185) were obtained from American Type Culture Collection (ATCC, Manassas, VA, USA). 16HBE14o-cells were cultured in Minimum Essential Modified Eagle’s Medium (Invitrogen) with 10% fetal bovine serum (FBS), while HeLa S3 cells were cultured in Minimum Essential Modified Eagle's Medium (Thermo Fisher Scientific, Waltham, MA, USA) with 2 mM l‐glutamine (MilliporeSigma, Burlington, MA, USA), antibiotics (100 U/ml of penicillin and 100 µg/ml of streptomycin (MilliporeSigma) with 10% FBS. A549 cells were cultured in RPMI 1640 media supplemented with 10% FBS in a humidified incubator at 37 °C in 5% CO_2_. All experiments were conducted at a final confluence of 70–80%.

### Chemicals

**Fig. 1:**
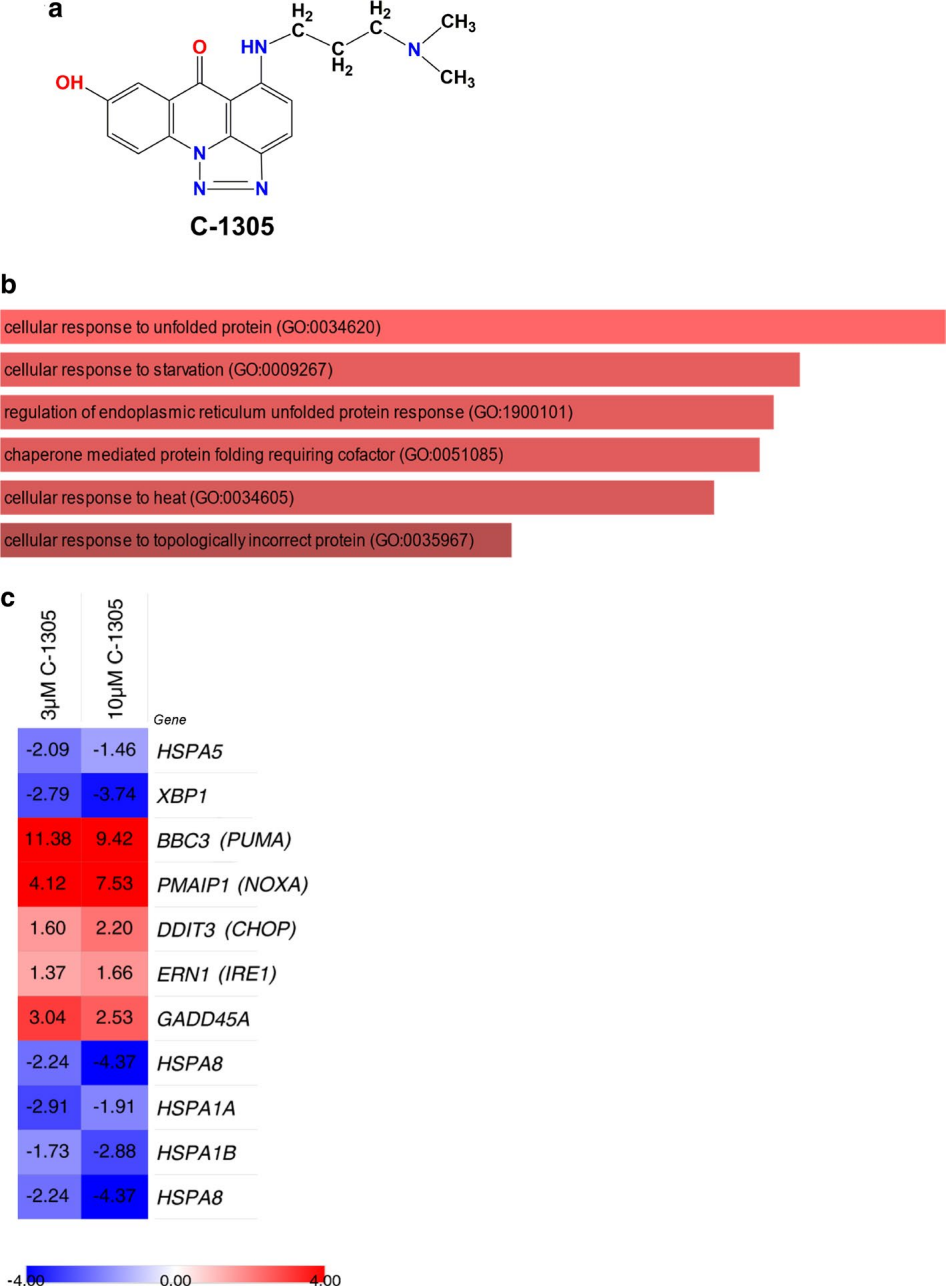
The NGS and pathway analyses of early gene transcripts dysregulated by C-1305 treatment in A549 cells. **a** The 2D chemical structure of C-1305 (5-((3-(dimethylamino)propyl)amino)-8-hydroxy-6H-[1,2,3]triazolo[4,5,1-de]acridin-6-one; C_18_H_19_N_5_O_2_). **b** The unique early gene transcripts dysregulated after 8 h of treatment of A549 with 3 µM C-1305 were selected from the NGS experiments. Well-established gene transcripts with greater than 10 RPKMs per sample and with significance (p ≤ 0.05) greater for change in expression between C-1305-treated and control groups (no treatment and 24-h treatment) were used in pathway analyses. The Gene Ontology clusters are depicted for selected genes; clusters are listed followed by the p values and enrichment scores calculated by Enricher, which is used to determine the percentage of the chart. The longer bar the lower p-value, while the darker the color, the more enriched the cluster. Only clusters with p ≤ 0.001 were considered. **c** The heat map representing C-1305 exposure related expression changes in genes related to UPR and ER stress as observed in NGS experiments in A549 cells exposed to 3 µM and 10 µM C-1305 for 8 h. Heat maps were generated with the Morpheus software (Morpheus, https://software.broadinstitute.org/morpheus). The color scale and values depict fold change (**c**)

5-{[3-(Dimethylamino)propyl]amino}-8-hydroxy-6H-[1,2,3]triazolo[4,5,1-de]acridin-6 one (C_18_H_19_N_5_O_2_, M_W_ = 337.38 g/mol) (compound C-1305) was synthesized by the Faculty of Chemistry, University of Gdansk, Poland, and verified for purity and identity with RP-HPLC, elemental analysis, ^1^H NMR spectroscopy and HR MS spectrometry as previously described in [21, 23, 24]. The C-1305 chemical formula is provided in Fig. 1a. Two independent batches of C-1305 were tested. C-1305 was stored in the dark at 4 °C. Prior to the experiments, the compound was always freshly dissolved in DMSO as a 3 mM stock solution. DMSO (Catalog no. S-002-D) was purchased from Sigma-Aldrich. The IRE1 activity inhibitor, 4µ8C, was purchased from Sigma-Aldrich (SML0949) and dissolved in DMSO prior to use.

### Induction of ER stress and activation of the UPR

Pharmacological induction of ER stress and activation of the UPR were performed as we previously described [25]. Briefly, cells were treated with the compounds for the time periods specified: tunicamycin (0.5 μg/mL; Sigma, T7765) and thapsigargin (Tg, 25 nM Sigma, T9033). Control (CTRL) cells were treated with vehicle CTRL, DMSO (< 0.5% v/v; Sigma, D2650).

### Isolation of RNA

Total RNA (containing both mRNA and miRNA) was isolated using miRNeasy kit (Qiagen). RNA concentrations were calculated based on the absorbance at 260 nm. RNA samples were stored at − 70 °C until use.

### Next‐generation RNA sequencing analyses

Cells treated with C-1305 or DMSO vehicle (as specified in “Results” section) were used for the RNA isolation and analyses. Following rRNA depletion, the remaining RNA fraction was used for library construction and subjected to 75-bp single-end sequencing on an Illumina NextSeq 500 instrument (San Diego, CA, USA). Sequencing reads were aligned to the Gencode human reference genome assembly (GRCh38 p7 Release 25) using STAR version 2.5.3b [26]. Transcript assembly and estimation of the relative abundance and tests for differential expression were carried out with HTSeq-count version 0.9.1 and DESeq2 for those samples with biological replicates, and with Cufflinks and Cuffdiff for those samples without biological replicates [26–28]. The resulting data were validated with quantitative real-time PCR. The heat map generation and hierarchical clustering were performed with the Morpheus Web server (Morpheus, https://software.broadinstitute.org/morpheus). The Enrichr Web server (https://amp.pharm.mssm.edu/Enrichr/) [29] was applied to assign the NGS results into the ‘Gene Ontology Biological Process’ categories with the selection based on a p‐value below p = 0.05. Furthermore, the analyses were limited to experimentally verified interactions and no extended gene enrichment set analyses were performed.

### Measurement of mRNA quantitative Real Time PCR (qRT-PCR)

We used TaqMan One-Step RT-PCR Master Mix Reagents (Applied Biosystems) as described previously [30] using the manufacturer’s protocol (retrotranscription: 15 min, 48 °C). The relative expressions were calculated using the 2^−ΔΔCt^ method [31] with the Glyceraldehyde 3-phosphate dehydrogenase (*GAPDH*—Hs02786624_g1) and (*TBP* Hs00427620_m1) genes as reference genes for the mRNA. TaqMan probes ids used: *HSPA5*—Hs00607129_gH, *BBC3* (*PUMA*)—Hs00985031_g1, *DDIT3* (*CHOP*)—Hs00358796_g1, *ERN1* (*IRE1*)—Hs00980095_m1, *PMAIP1* (*NOXA*)—Hs00560402_m1, *XBP1* (isoform 1and 2)—Hs00231936_m1, *XBP1s*—Hs03929085_g1.

### Western blots

XBP1s protein detection was performed as described in [32]. Briefly, cells were lysed on ice for 15 min in RIPA buffer [150 mM NaCl, 1% NP‐40, 0.5% sodium deoxycholate, 0.1% SDS, and 50 mM Tris‐HCl (pH 8.0)] supplemented with Protease Inhibitor Complete Mini (000000011836170001; Roche, Basel, Switzerland). The insoluble material was removed by centrifugation at 15,000*g* for 15 min. Protein concentrations were determined by Bio‐Rad Protein Assay (Bradford‐based method; Bio‐Rad, Hercules, CA, USA) using bovine serum albumin (BSA; MilliporeSigma) as the standard. Following the normalization of protein concentrations, lysates were mixed with an equal volume of 2× Laemmli sample buffer (Bio‐Rad) and incubated for 5 min at 95 °C before separation by SDS‐PAGE on stain‐free TGX gradient gels (Bio‐Rad). Following SDS‐PAGE, the proteins were transferred to PVDF membranes (300 mA for 90 min at 4 °C). The membranes were then blocked with BSA dissolved in PBS and Tween‐20 (3% BSA and 0.5% Tween‐20 for 1–2 h) followed by immunoblotting with the primary antibody for each experiment for XBP1s (mAb 12782; diluted at 1:1000; Cell Signaling Technology, Danvers, MA, USA). After the washing steps, the membranes were incubated with goat anti‐rabbit IgG (H + L) horseradish peroxidase-conjugated secondary antibodies (Bio‐Rad) and detected using ECL (Amresco, Solon, OH, USA). Densitometry was performed using Image Lab software v.4.1 (Bio‐Rad).

### Molecular docking

The crystal structure of human phosphorylated IRE1α in complex with ADP-Mg (PDB ID code 4YZD, RSCB Protein Data Bank) [33] was used for molecular docking performed in this study. The structure of IRE1 was prepared for docking analysis on the UCSF Chimera software package [34] where the multi-chain structure was converted to a monomeric structure (chain A), and water and ADP-Mg were deleted to enable testing of studied ligand binding. A mol2 file with all hydrogens and 3D coordinates of C-1305, 4µ8C IRE1 [35], and 3,6 DMAD (9-*N*-[3-(dimethylamino)propyl]-3-*N*,3-*N*,6-*N*,6-*N*-tetramethylacridine-3,6,9-triamine) [36] IRE1 inhibitors were generated using MarvinSketch (ver. 20.18.0 ChemAxon Ltd) [36]. Energy optimizations of C-1305, 4µ8C, and 3,6-DMAD with 500 steps of the steepest descent algorithm using the MMFF94 force field were carried out for all 2D structures using the Avogadro 1.2.0 package software [37]. The minimized structures were used as inputs for ligand binding modeling. Furthermore, all three compounds were docked into their potential binding pockets of the native 3D structure of IRE1 proteins (PDB ID code 4YZD) with the aid of EDock that is based on replica-exchange Monte Carlo simulations that identifies high-quality blind docking sites [38]. Only protein fragments that are not well ordered in the crystal structure were predicted using the I-TASSER (Iterative Threading ASSEmbly Refinement), a part of EDock server. This simulation does not disturb the native IRE1 binding pocket structure. RMSD (UCSF Chimera) between 143 pruned atom pairs is 0.000 angstroms (across all 143 pairs: 0.000). For the docking analyses, only the best scoring results were used. The results of docking were visualized with UCSF Chimera.

### In vitro kinase assay

ERN1 (IRE1α) Kinase Enzyme assays were performed using the luminescent ADP-Glo™ assay kit (VA7146, Promega) that measures the generation of ADP by the protein kinase reaction that is measured by an increase in the luminescence signal in the presence of luminescent ADP-Glo™ reagent. All reagents were prepared according to the manufacturer’s instructions. To determine the appropriate enzyme concentration for use in each inhibitor dose–response curve determination, a kinase titration was performed using 10 µM ATP. Based on these results, a concentration of 5 ng of IRE1 enzyme per reaction was chosen to determine the kinase inhibitory activity of C-1305 and 4µ8C (SML0949, Sigma-Aldrich). Staurosporine (S6942-200UL, Sigma-Aldrich) was used as a control kinase inhibitor. All reactions were measured using the GloMax^®^-Multi Detection System (Promega). The results were expressed as remaining activity, normalized to the uninhibited control for 100% activity and to the fully inhibited one for 0% activity. To normalize the data from the following equation was used:

$$''\% {\text{ Remaining activity}} = {1}00 \times \left( {{\text{S}} - {\text{B}}/{\text{U}} - {\text{B}}} \right)",$$

"S" being the signal obtained for each concentration of compound, "U" the signal produced by the uninhibited control (enzyme alone), and "B" the background produced by the fully inhibited control (no enzyme). The data were plotted and fit to sigmoidal curves using Origin Pro ver 7.0 (OriginLab Corporation, Northampton, MA, USA) to determine the IC_50_.

### Statistical analysis

Results were expressed as a mean ± standard deviation. Statistical significance was determined using the Student’s t-test and ANOVA on ranks with p-values below *p* = *0.05* considered significant.

## Results

We previously utilized a genome-wide approach to identify transcriptomic signatures of C-1305 cytotoxicity in lung cell lines and demonstrated that C-1305 promotes direct microtubule stabilization as a part of its mechanism of action that leads to apoptosis [21]. We also observed that C-1305 at concentrations up to 10 µM had no significant effect on growth and survival of an adenocarcinoma lung cell line, A549, up to about 18 h [21]. To further evaluate the early transcriptomic signaling that contributes to C-1305’s biological effects, we focused on the genome-wide consequences of 8 h exposure of A549 cells to 3 µM C-1305, a concentration that did not affect cell survival during this time of exposure [21]. In this experiment, total RNA was extracted from the A549 cells that were exposed to C-1305 for 8 h and from 8 h DMSO vehicle-treated cells (controls), and then subjected to RNA sequencing. To focus only on the C-1305 early transcriptome changes, we corrected (subtracted out) the A549-C-1305-8 h treated gene set with the A549-8 h vehicle-treated gene set and also the previously obtained A549-C-1305-24 h treated gene set [21]. Our analysis was narrowed to the most significant expression changes (p ≤ 0.05). This approach resulted in the identification of 182 genes dysregulated specifically at the C-1305 treatment 8 h time point and these genes were further subjected to gene ontology analysis using Enricher [29]. As shown in Fig. 1b, the cellular response to unfolded/misfolded proteins was the most significant assignment. Within this cluster of gene transcripts, the following genes’ mRNA levels were downregulated upon C-1305 exposure: heat shock protein family A member 5 (*HSPA5* aka *BiP*), *XBP1*, heat shock protein family A member 8 (*HSPA8*), heat shock protein family A member 1A (*HSPA1A*) and heat shock protein family A member 1B (*HSPA1B*) (Fig. 1c). Notably, the upregulated genes in this set were Bcl-2 binding component 3 (*BBC3* aka *PUMA*), phorbol-12-myristate-13-acetate-induced protein 1 (*PMAIP1* aka NOXA), activating transcription factor 3 (*ATF3*), DNA damage inducible alpha (*GADD45A*) as well as *ERN1* (aka *IRE1*) and DNA damage inducible transcript 3 (*DDIT3* aka *CHOP*) (Fig. 1c). Notably, similar and even more dramatic transcriptomic changes were also observed up on 8 h exposure to 10 µM concentration of C-1305 (Fig. 1c).

Based on the results of the RNAseq-based approach, we speculated that C-1305 may impair endoplasmic reticulum (ER) protein homeostasis via downregulation of transcripts of chaperone (BiP) and proadaptive transcription factors (XBP1s) and this could lead to activation of UPR pathways. Furthermore, the UPR proapoptotic factors like CHOP, PUMA, NOXA and GADD45A were elevated at this time. To verify this observation, we performed qPCR analysis of these UPR related transcripts levels after 8 h C-1305 treatments at 1 µM, 3 µM and 10 µM concentrations in A549 as well as in a non-cancer lung cell line 16HBE14o-, and in HeLa cells. We included HeLa cells since this cell line is a well recognized and characterized model for studying pharmacological ER stress and UPR induction, including the IRE1/XBP1s branch [39–47].

**Fig. 2:**
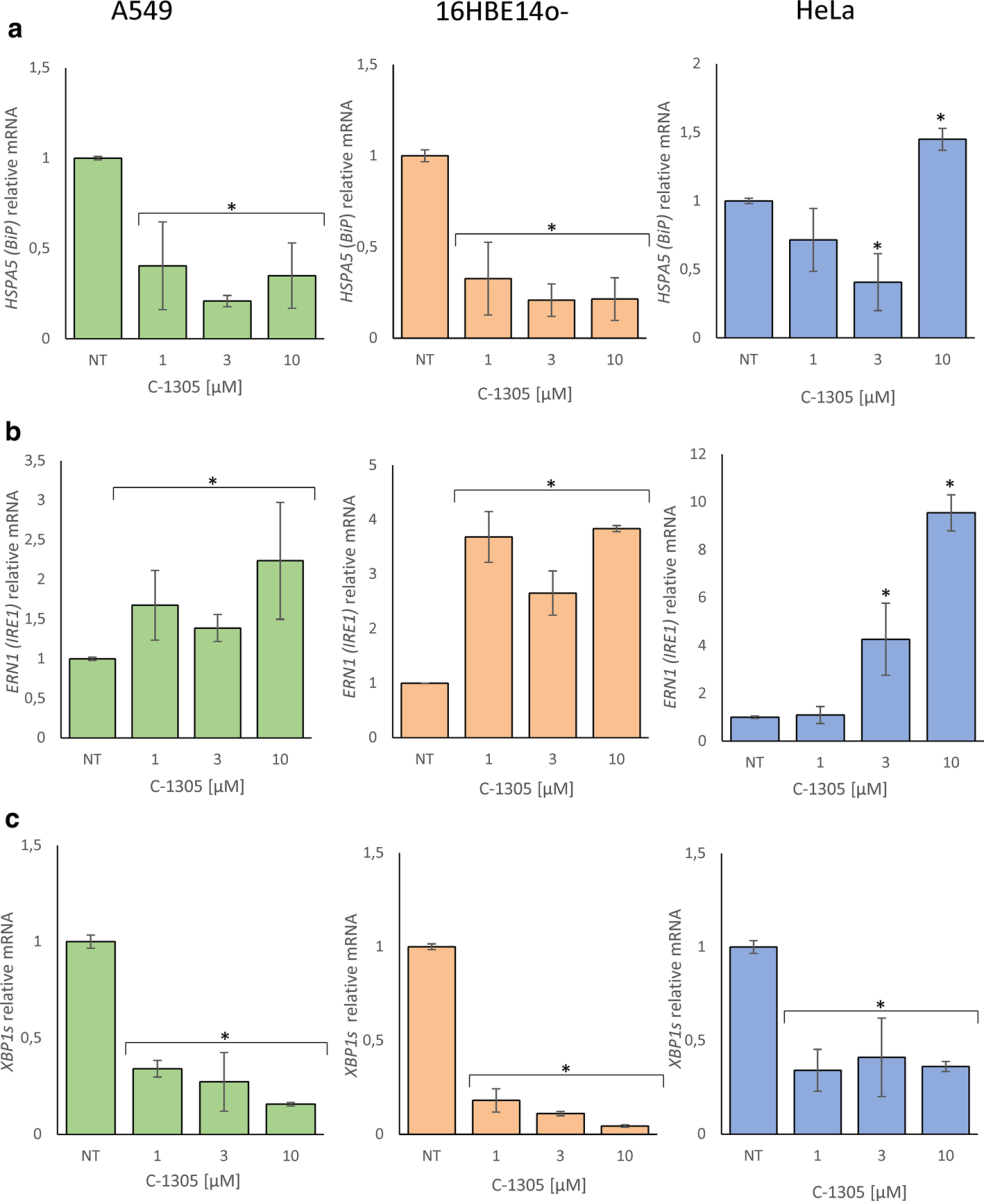
C-1305-induced changes in **a** *BiP*, **b** *ERN1*, **c** *XBP1s* mRNA levels in A549, 16HBE14o- and HeLa cells after 8 h of treatment. The results from 3 independent experiments (n = 9) are plotted normalized to *GAPDH* and *TBP* mRNA levels and expressed as a fold-change over the vehicle controls. Error bars represent standard deviations. Significant changes (*p-value* < 0.05) are marked with an asterisk

As shown in Fig. 2, the 8 h C-1305 exposure of all cell lines significantly reduced *BiP* expression and resulted in significant induction of *ERN1* mRNA levels. Although, the cell lines differed between each other in terms of C-1305 dose response dependence and magnitudes of transcriptomic changes, the general compound’s effects on *BiP* and *ERN1 (IRE1)* mRNA levels correlated fairly well with the C-1305 concentrations except for the 10 µM concentration in the HeLa cells. In examining the effects of C-1305 on the active isoform of *XBP1, XBP1s*, the results indicated that the levels of this mRNA were dramatically lower in all three cell lines (Fig. 2c) which was surprising given that the *ERN1* mRNA levels were increased.

**Fig. 3:**
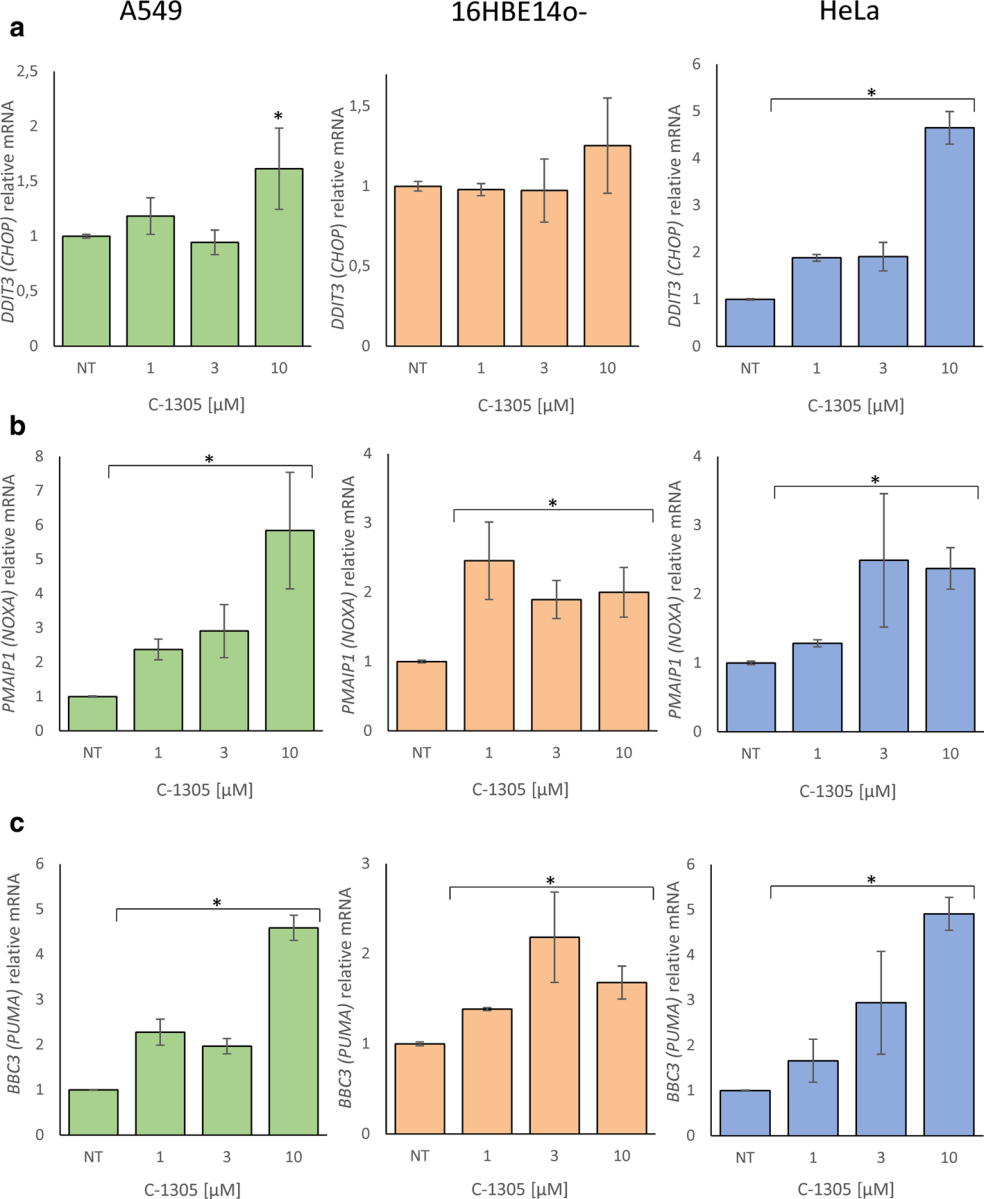
C-1305-induced changes in **a** *CHOP*, **b** *NOXA*, **c** *PUMA* mRNA levels in A549, 16HBE14o- and HeLa cells after 8 h of treatment. The results from 3 independent experiments (n = 9) are plotted normalized to *GAPDH* and *TBP* mRNA levels and expressed as a fold-change over the vehicle controls. Error bars represent standard deviations. Significant changes (*p-value* < 0.05) are marked with an asterisk

In terms of the proapoptotic factors, there was no significant induction of *CHOP* observed in airway cells, 16HBE14o- and A549, after exposure to 1 µM and 3 µM C-1305, whereas it was in the HeLa cells at all three concentrations (Fig. 3a). Nevertheless, a moderate *CHOP* induction was observed in the NGS analysis of A549 exposed to 3 µM C-1305, however, this discrepancy results from the different gene expression normalization methods applied for NGS and qPCR data (global—DESeq2 and individual—*GAPDH* and *TBP* reference genes, respectively). In contrast, there was a significant induction of both proapoptotic *NOXA* and *PUMA* mRNA expression at all three concentrations in all of the tested cell lines (Fig. 3b, c). The collective data in Figs. 2 and 3 suggest that C-1305 treatment induced IRE1, but paradoxically, decreased *XBP1s* mRNA expression, and that for the most part, the UPR prosurvival genes decreased and the proapoptotic genes increased.

**Fig. 4:**
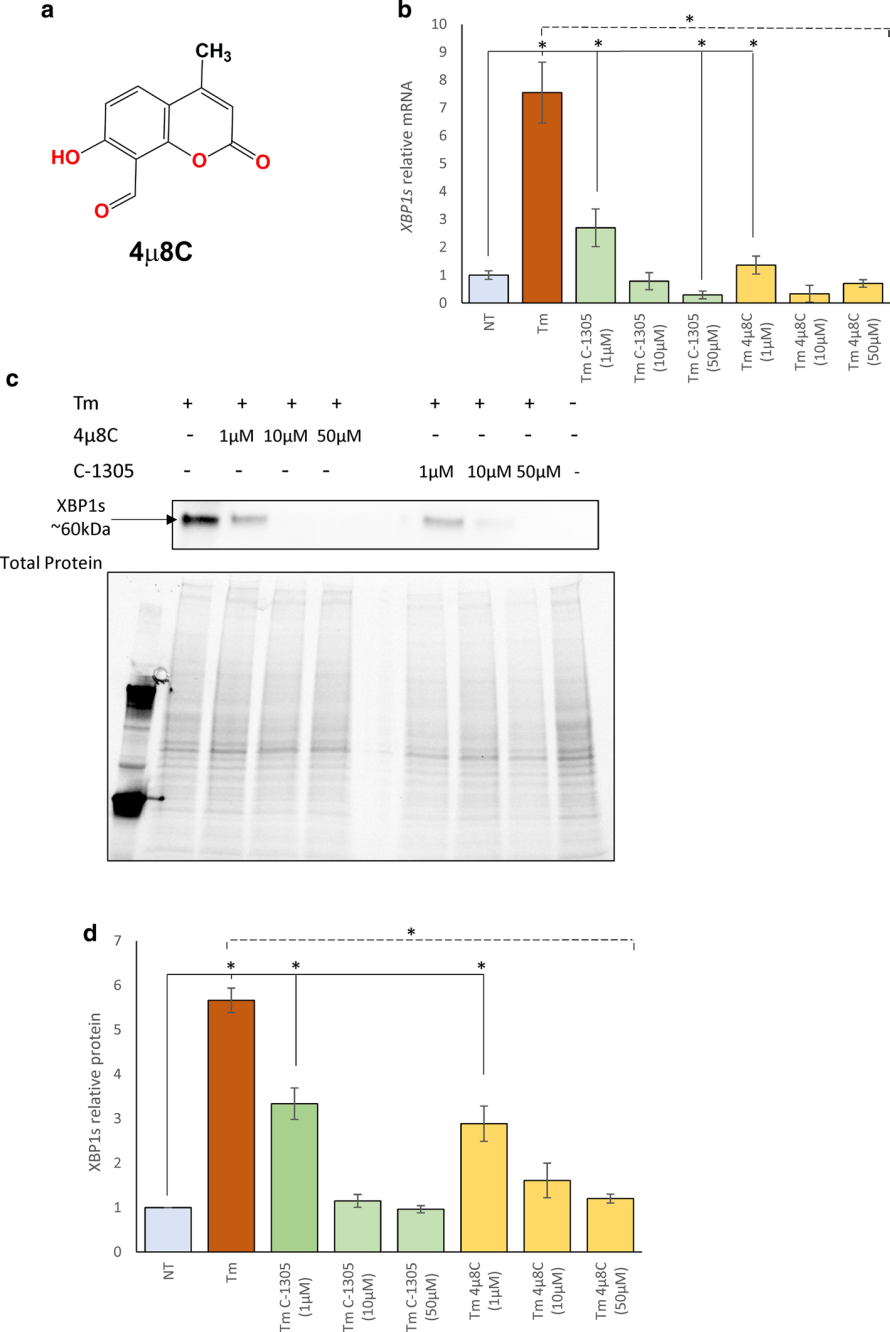
C-1305-prevents IRE1α-dependent *XBP1* mRNA splicing during ER stress. **a** The 2D chemical structure of 4µ8C (7-Hydroxy-4-methyl-2-oxo-2H-chromene-8-carbaldehyde, C_11_H_8_O_4_). **b** HeLa cells were treated with ER stressors (Tm, 0.5 µg/ml) for 6 h in a presence of specified concentrations of C-1305 and 4µ8C. Following the treatments, the *XBP1s* mRNA levels were accessed with qPCR and expressed as a fold-change over the no stress controls. The results from 3 independent experiments (n = 9) are plotted normalized to *GAPDH* and *TBP* mRNA. Error bars represent standard deviations. Significant changes (*p-value* < 0.05) are marked with an asterisk. **c** The corresponding changes in XBP1s protein levels were evaluated by Western blot (**d**) normalized to total protein levels and related no stress control. The data from 3 independent experiments were analyzed. *p < 0.05 was considered significant. The raw data are provided in Additional file 1

Based on these results, we next tested whether the decrease in *XBP1s* was due to a inhibition of IRE1α endoribonuclease activity. We compared the C-1305 effectiveness in preventing IRE1α-dependent *XBP1* mRNA splicing with the efficiency of commonly used IRE1 inhibitor 4µ8C [35, 48] (Fig. 4a). As an ER stress induced *XBP1* mRNA splicing model, we used HeLa cells treated for 6 h with the classical pharmacological stressor tunicamycin (Tm, 0.5 ug/ml), a glycosylation inhibitor. We used HeLa cells for this experiment since they are commonly used in ER stress and UPR studies [39–43]. Previous studies using this model indicated that maximal *XBP1s* mRNA levels are observed after 6 h of treatment [44]. Furthermore, both treatments of HeLa cells with C-1305 for 6 h or co-treatments with Tm and Tg had a limited effect on cell survival (IC_50_ = 37,62 µM for C-1305, IC_50_ = 34,80 µM for C-1305 and Tm; IC_50_ = 21,6 µM for C-1305 and Tg; as monitored by the MTT assay, Additional file 1: Figure S1).

As shown in Fig. 4b, the use of Tm resulted in about a sevenfold induction of *XBP1s* mRNA, whereas use of 1 µM, 10 µM and 50 µM of C-1305 resulted in dramatic reduction of XBP1s mRNA levels and appeared very similar to the Tm 4µ8C treatments. Furthermore, the XBP1s protein reduction was similar for both the C-1305 and 4µ8C (Fig. 4c, d), suggesting that C-1305 inhibited the IRE1 endoribonuclease activity and the subsequent production of XBP1s.

**Fig. 5:**
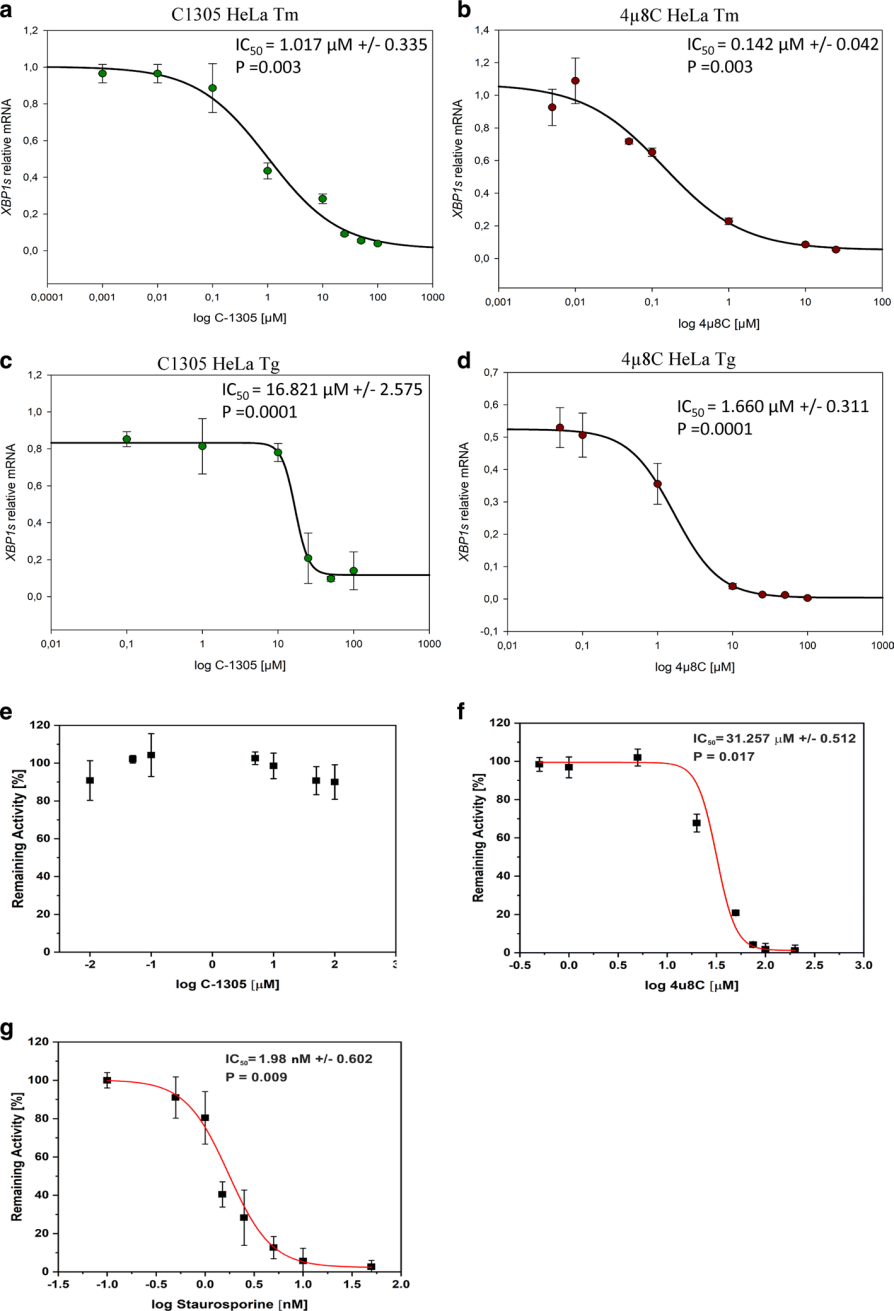
The *XBP1s* mRNA-based determination of C-1305 impact on IRE1α endoribonuclease activity in ER stressed HeLa cells. HeLa cells were treated with ER stressors (Tm, 0.5 µg/ml and Tg, 25 nM), for 6 h in a presence of specified concentrations of C-1305 and 4µ8C. Following the treatments, the *XBP1s* mRNA levels were accessed with qPCR and expressed as a change versus ER stressed cells treated with vehicle. The results from 3 independent experiments (n = 9) are plotted normalized to *GAPDH* and *TBP* mRNA. Error bars represent standard deviations. Concentration response curves and IC_50_ values of **a** C-1305 and **b** 4µ8C in Tm treated cells were determined using Sigma Plot 1.1 software. Similar approach was used to determine Concentration response curves and IC_50_ values of **c** C-1305 and **d** 4µ8C in Tg treated cells. The IRE1α kinase assays were performed in the presence of serial dilution and were measured with ADP-Glo kinase assay. Concentration response curve and IC_50_ values of **e** C-1305, **f** 4µ8C and **g** staurosporine (used as a control of kinase activity) were determined using Origin Pro software. The results were expressed as remaining activity, normalized to the uninhibited control for 100% activity and to the fully inhibited one for 0% activity as described in “Materials and methods”. Data are plotted as means ± SD from triplicate measurements of two independent measurements (n = 6)

To characterize the C-1305 as a potential IRE1 inhibitor, we used the HeLa model to determine C-1305’s IRE1α endoribonuclease activity IC_50_ values based on the measurement of *XBP1s* mRNA levels and used 4µ8C as our control. As shown in Fig. 5a, in Tm treated HeLa cells, the IC_50_ for C-1305 was 1.017 µM, whereas the IC_50_ for 4µ8C under the same conditions was 0.142 µM (Fig. 5b). Next, we tested another common ER stress model thapsigargin (Tg, 25 nM), a noncompetitive inhibitor of the endoplasmic reticulum Ca^2+^ ATPase that results in higher induction of *XBP1s* mRNA [23, 32]. In this case, higher concentrations of both C-1305 and 4µ8C were necessary to reduce the *XBP1s* mRNA levels (IC_50_ = 16.821 µM for C-1305 and IC_50_ = 1.66 µM for 4µ8C, Fig. 5c, d). Taken together, the results suggest that C-1305 is an efficient inhibitor of IRE1α and this compound works independently of the mechanism of pharmacological ER stress induction.

Using purified IRE1 protein, we next determined C-1305’s and 4µ8C’s effects on the IRE1α kinase activity in vitro and used staurosporine as a reference control. As shown in Fig. 5e, the C-1305 has no effect on IRE1α kinase activity, whereas 4µ8C was able to inhibit half of IRE1 phosphorylation at 31.25 µM concentration (Fig. 5f). These results suggest that C-1305 mechanism of action is based on inhibiting IRE1α endoribonuclease activity rather than kinase activity, whereas 4µ8C can block both endoribonuclease and kinase activity of IRE1. That being said, 4µ8C IC_50_ value for the kinase activity is remarkably high when compared with staurosporine (1.98 nM, Fig. 5g), and significantly higher than the concentrations required to reduce the endoribonuclease activity of IRE1α based on the *XBP1s* message readout.

**Fig. 6:**
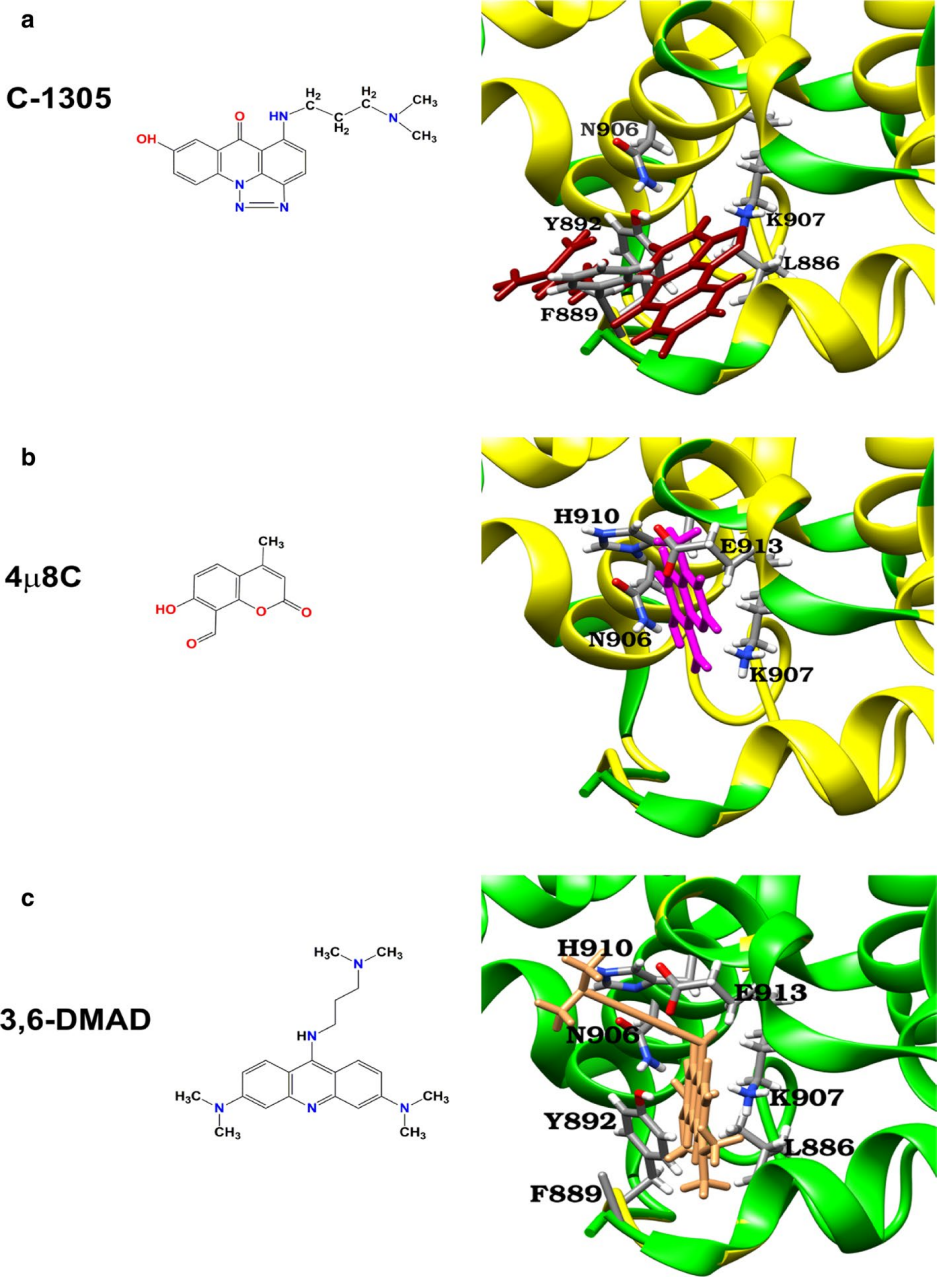
Prediction of C-1305, 4µ8C, and 3,6-DMAD binding mode in the and in the endoribonuclease (RNase) domain of IRE1α. The binding mode of **a** C-1305, **b** 4µ8C, and **c** 3,6-DMAD was modeled using the EDock server (https://zhanglab.ccmb.med.umich.edu/EDock/). The native and modeled structures of the IRE1 chain A are represented in green and yellow, respectively. ADP structure is represented by red, C-1305 by red sticks, 4µ8C by pink sticks, and 3,6-DMAD by sandy brown sticks. Predicting protein–ligand binding residues are represented by grey sticks together with oxygen, nitrogen, sulfur, and hydrogen atoms (colored red, blue, yellow, and white, respectively) and numbered according to IRE1α sequence **(**PDB ID code 4YZD). Magnesium ion (Mg) is colored by cornflower blue

Since our results suggested that C-1305 may inhibit IRE1α via direct interaction with its RNAse domain, we evaluated docking of C-1305 targeting the catalytic site of the RNase domain using molecular docking analysis (Fig. 6). The model of IRE1α in complex with C-1305 inhibitor obtained in the docking analysis suggests that this molecule can form hydrophobic interactions with F889 and L886 residues, a hydrogen bond with Y892 and N906 (Fig. 6a). Molecular docking results clearly suggest that triazole ring can interact with both Lysine via H-bonding and Leucine via a pi-alkyl interaction. These interactions could stabilize C1305 in the ribonuclease active site of IRE1α. In contrast, the docking of 4µ8c to IRE1α shows interactions with H910, N906, K907, and E913 and was similar to the previous docking model published by [35], Hanwell, Curtis [37] indicated that Y892, F889, N906, K907, and H910 residues of IRE1α interact with 4µ8c (Fig. 6b). The mode of action of 4µ8c is related to the formation of a Schiff base with K907 residue. Despite the fact that C1307 does not have an aldehyde group, it is conceivable that it forms an imine bond with K907 due to the presence of a carbonyl group in its structure. Since structurally C-1305 is related to a known IRE1α inhibitor, acridine derivative 3,6-DMAD [36], we also prepared a docking model of 3,6-DMAD with IRE1α (Fig. 6c) and showed the interaction of 3,6-DMAD with H910, N906, K907, L886, Y892, F889, and E913 residues of IRE1α that are present in the catalytic site of the RNAse domain of IRE1α. Taken together, the results of the computational modeling strongly support the hypothesis that C-1305 is a direct IRE1α inhibitor, however, further experimental studies will be required to confirm these findings.

## Discussion

The IRE1α-dependent pathway has been considered as a one of main transducers of the UPR and an important determinant of cell fate [49–51]. Despite the fact that IRE1α’s molecular activities are mostly related to facilitating cellular survival, during irrevocable ER stress, this enzyme can also contribute to accumulation of proinflammatory and apoptotic factors [52]. Thus, gaining control over IRE1α’s activities with small molecules remains a promising approach to improving cancer treatments by inhibiting IRE1α activity during the early stages of tumorigenesis [53–55] and by preventing tumor progression and metastasis either by utilizing proapoptotic abilities of this enzyme [4] or inducing apoptosis through other signaling pathways such as PERK. Nevertheless, most studies focus on inhibiting IRE1α activity to impair the adaptation of tumor cells to their cellular stressors. Currently, numerous compounds has been shown to be highly selective in preventing IRE1α kinase, RNAse or both activities and are characterized by EC_50_ values ranging from 0.02 to 20 µM (reviewed in [4]). Furthermore, molecules such as astoyocamycin, doxorubicin, quinotrierixin, and trierixin were shown to inhibit IRE1α/XBP1s activity in vitro and in vivo despite no clear determination of their mechanism of their action with IRE1α [4]. Although some structural similarities can be found among known IRE1α inhibitors, their number remains limited, and many of the compounds have been identified in high-throughput screens [4]. Hence, identification of novel structural motifs responsible for preventing IRE1α kinase or RNAse activity may promote a more rational design and development of better IRE1α inhibitors.

In this study, based on observations from an unbiased transcriptomic screen for molecular mechanisms of action of cytotoxic triazoloacridone C-1305 in cancer cell lines [21], we identified that a known topoisomerase inhibitor and microtubule-stabilizing agent can also inhibit XBP1s activity, presumably by inhibiting the endoribonuclease activity of IRE1α. Our studies indicate that C-1305 treatment reduces *XBP1s* mRNA levels and downregulation of ER protein folding machinery including BiP and the accumulation of ER stress-related apoptotic factors mRNAs including *NOXA* and *PUMA*. We also verified and compared C-1305 to 4µ8C, a well-established IRE1α endoribonuclease inhibitor, and found using XBP1s message as our readout that the IC_50_ for 4µ8C was 0.142 µM, which compares favorably to the literature range of 0.076 to 6.9 µM [35]).

Although we found that C-1305 had no kinase inhibitory activity, we did observe that 4µ8C did have a modest effect on kinase activity (IC_50_ of 31 µM). Although this compound was been initially assigned as an IRE1α RNAse inhibitor, our observation is consistent with previous reports of Cross, Bond [35] who demonstrated that 4µ8C can inhibit IRE1 autophosphorylation in cell-free assays via Schiff base formation with IRE1α K599 in the absence of ADP. However, this cellular nucleotide prevents 4u8C from targeting IRE1α K599 intracellularly [35].

Furthermore, our model of IRE1α in complex with C-1305 inhibitor obtained in the docking analysis strongly support the hypothesis that C-1305 is a direct IRE1α inhibitor, however, further experimental studies will be required to confirm these findings.

In conclusion, we identified C-1305 as potential inhibitor of IRE1α RNAse activity and previous studies reported this compound is potent cytotoxic agent and an atypical topoisomerase poison and microtubule stabilizing agent. C-1305 may provide a rational platform for further development of this class of compounds in anti-cancer therapies.

## Supplementary Information

**Additional file 1:** Evaluation of C-1305 cytotoxicity in HeLa cells exposed for 6 hours to different concentrations of C-1305 and ER stressors, and supplementary materials for Figure 4 (three examples of the uncropped Western blots and SDS Page gels used to follow XBP1s changes).

## Data Availability

Deep sequencing data were deposited in Gene Expression Omnibus (GEO) at Accession number: GSE143649.

## Abbreviations

UPR: Unfolded protein response
IRE1α: Inositol-requiring protein 1 alpha
PERK: Protein kinase RNA-like ER kinase
ATF6: Activating transcription factor 6
RIDD: Regulated IRE1-dependent decay
XBP1: X-box binding protein 1
XBP1s: Spliced XBP1
ERAD: ER associated protein degradation
Rnase: Endoribonuclease
