# Supplementary material for: Triazoloacridone C-1305 impairs XBP1 splicing by acting as a potential IRE1α endoribonuclease inhibitor
Source: Cell Mol Biol Lett. 2021 Mar 17;26:11. doi: 10.1186/s11658-021-00255-y (PMC7968329; doi:10.1186/s11658-021-00255-y)

# Additional file

# Triazoloacridone C-1305 impairs *XBP1* splicing being a potential IRE1α RNase activity inhibitor

Sylwia Bartoszewska^1^, Jarosław Króliczewski^2^, David K. Crossman^3^, Aneta Pogorzelska^4^, Maciej Bagiński^5^, James F. Collawn^6^ and Rafal Bartoszewski^2*^

^1^ Department of Inorganic Chemistry, Medical University of Gdansk, Gdansk, Poland.

^2^ Department of Biology and Pharmaceutical Botany, Medical University of Gdansk, Gdansk, Poland.

^3^ Department of Genetics, Heflin Center for Genomic Science, University of Alabama at Birmingham, Birmingham, USA, Birmingham, AL 35233.

^4^ Department of Organic Chemistry, Medical University of Gdansk, Gdansk, Poland.

^5^ Department of Pharmaceutical Technology and Biochemistry, Faculty of Chemistry, Gdansk University of Technology, 80-233 Gdansk, Poland

^6^ Department of Cell, Developmental and Integrative Biology, University of Alabama at Birmingham, Birmingham, USA, Birmingham, AL 35233.

***** Correspondence: Rafal Bartoszewski, Department of Biology and Pharmaceutical Botany, Medical University of Gdansk, Hallera 107, 80-416 Gdansk, Poland; Tel: 48 58 349 32 14; Fax: 48 58 349 32 11; email: [rafalbar@gumed.edu.pl](mailto:rafalbar@gumed.edu.pl); or Sylwia Bartoszewska Department of Inorganic Chemistry, Medical University of Gdansk, Hallera 107, 80-416 Gdansk, Poland; email: sylwiabart@gumed.edu.pl.


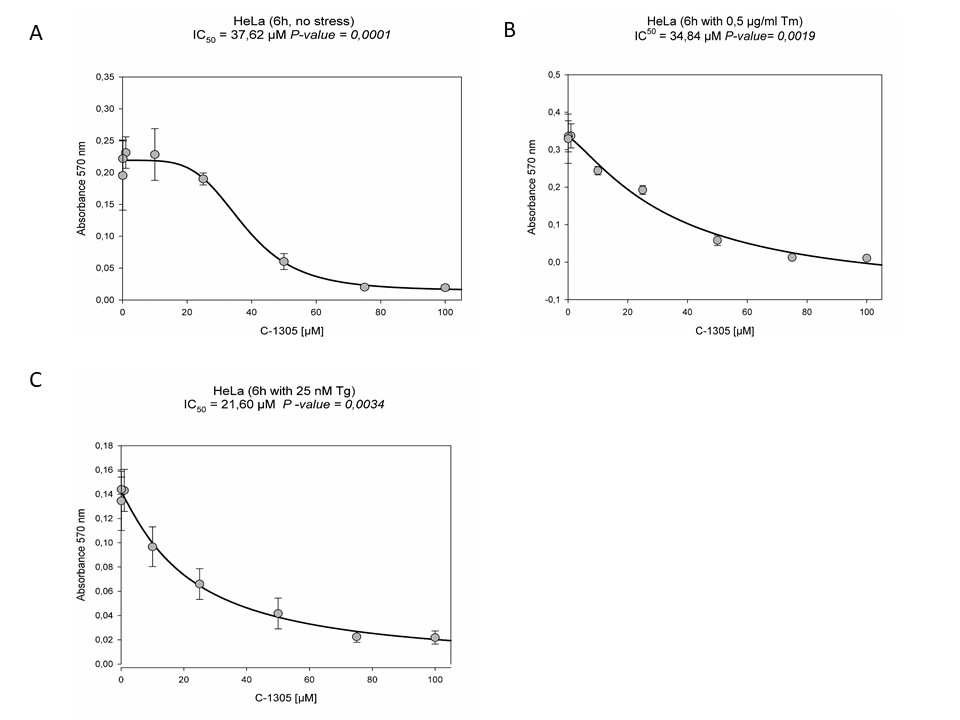


Figure S1. Evaluation of C-1305 cytotoxicity in HeLa cells exposed for 6 hours to different concentrations of C-1305 (A) and ER stressors: 0.5µg/ml Tm (B) or 25nM Tg (C). MTT assays of cell viability were performed in 96-well plates. One day after plating, HeLa cells (7,500 cells per well) were cultured with different concentrations of C-1305 in the presence or absence of ER stressors. After a 6-hour incubation with the specified compounds, medium containing 1 mg/mL MTT was added to cells for a final concentration of 0.5 mg/mL and incubated at 37 °C for 4 h. The medium was aspirated, and the formazan product was solubilized with DMSO. The absorbance at 630 nm (background absorbance) was subtracted from absorbance at 570 nm for each well. There were six replicates for each tested concentration. DMSO was used as control and the concentration in the medium did not exceed 0.1%. The IC_50_ values were calculated from dose–response curves using the four-parameter logistic function.

Additional file for Figure 4. Three examples of the uncropped Western blots and SDS Page gels used to follow XBP1s changes, in **Figure 4CD** of the manuscript:

Experiment 1. (Biological replicate 1).

Western blot


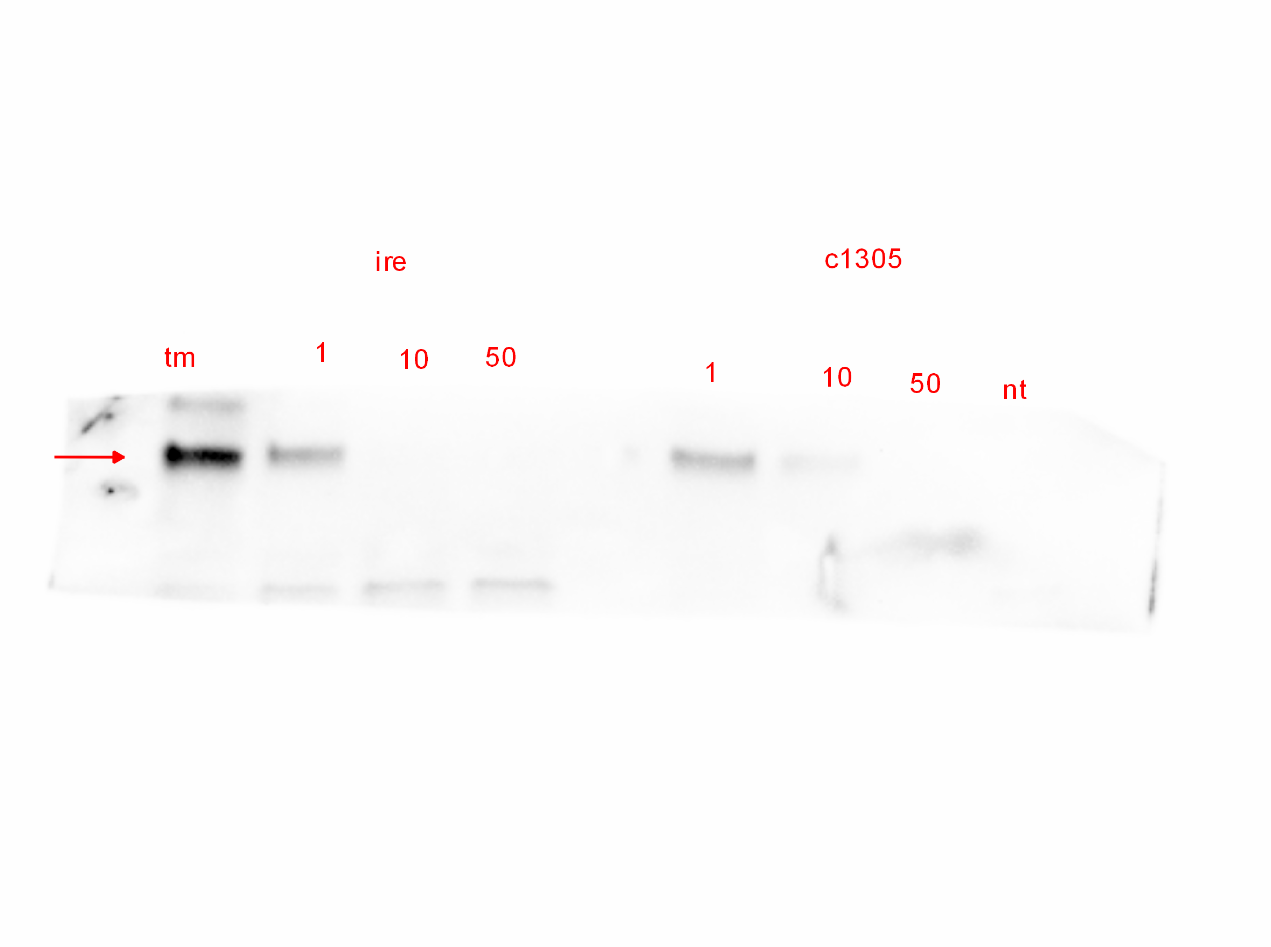


SDS Page gel – for total protein


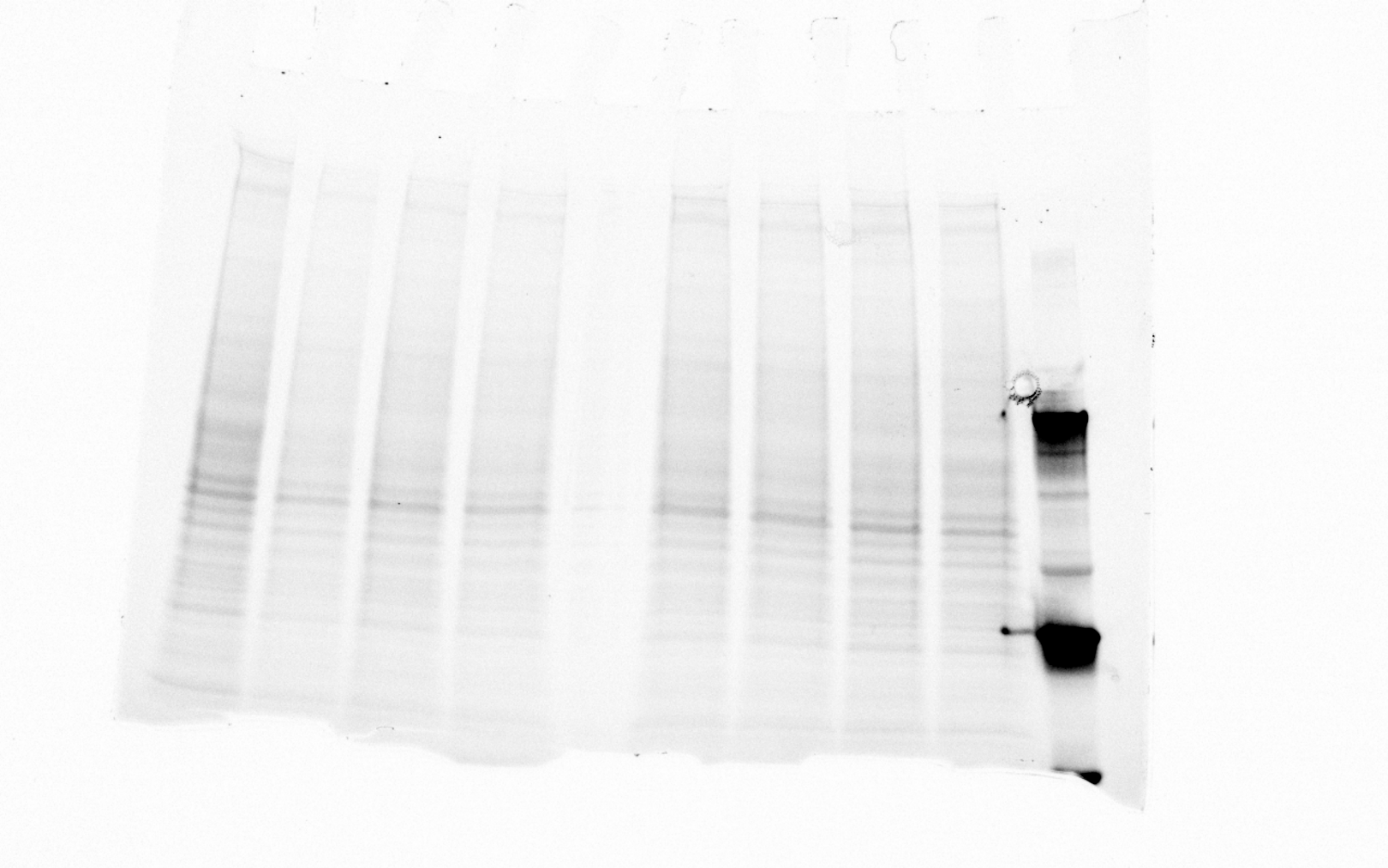


Experiment 2. (Biological replicate 2).

Western blot


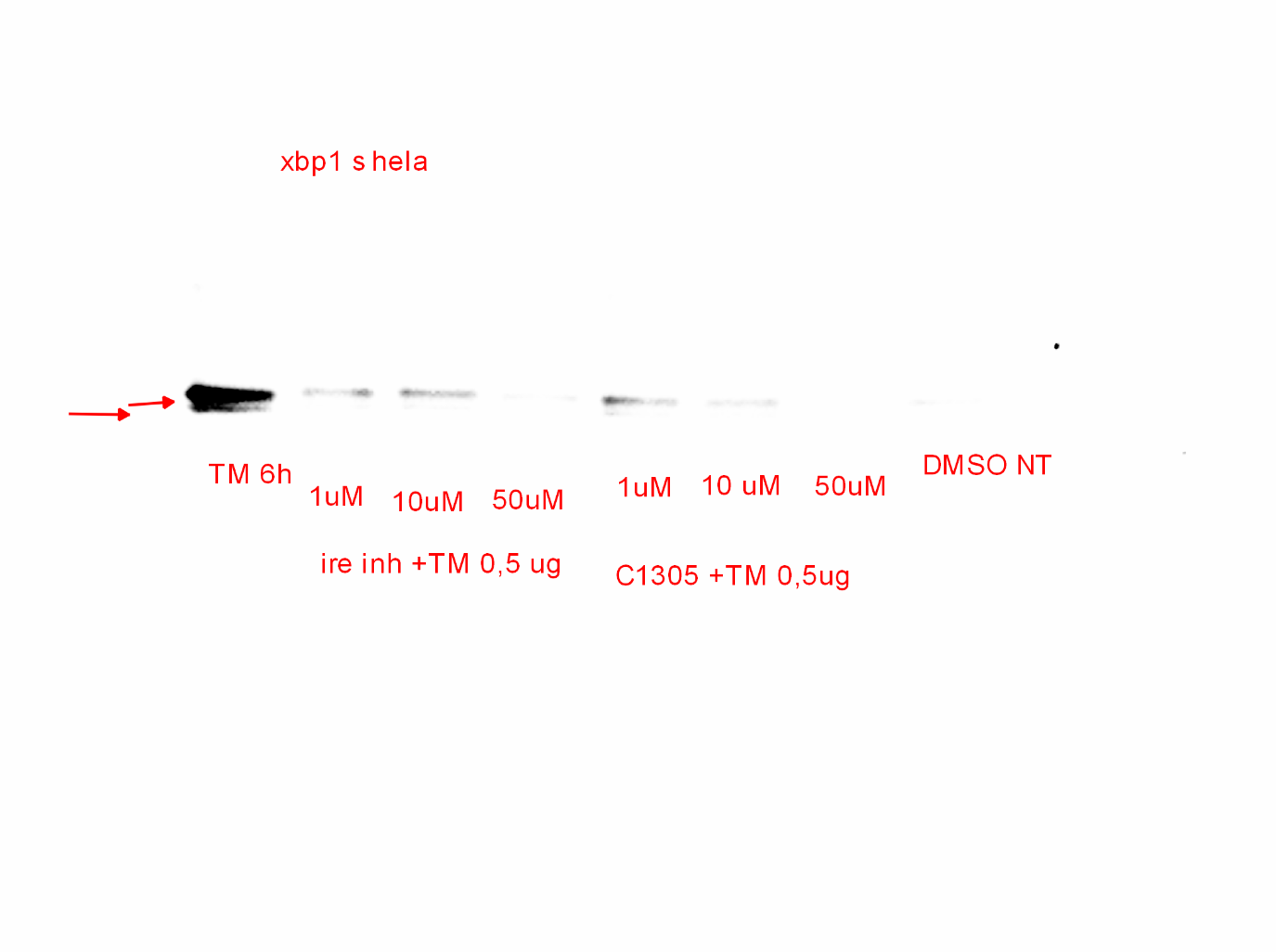


SDS Page


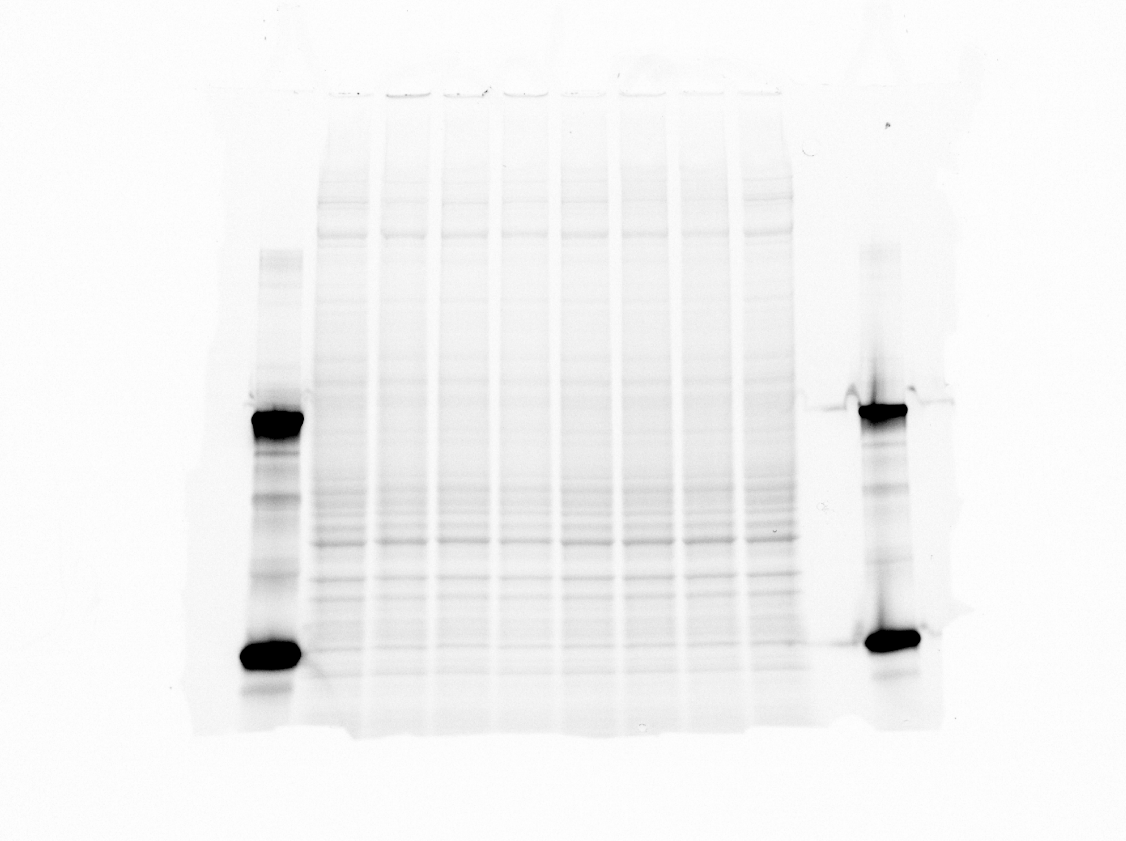


Experiment 3. (Biological replicate 3).

Western blot


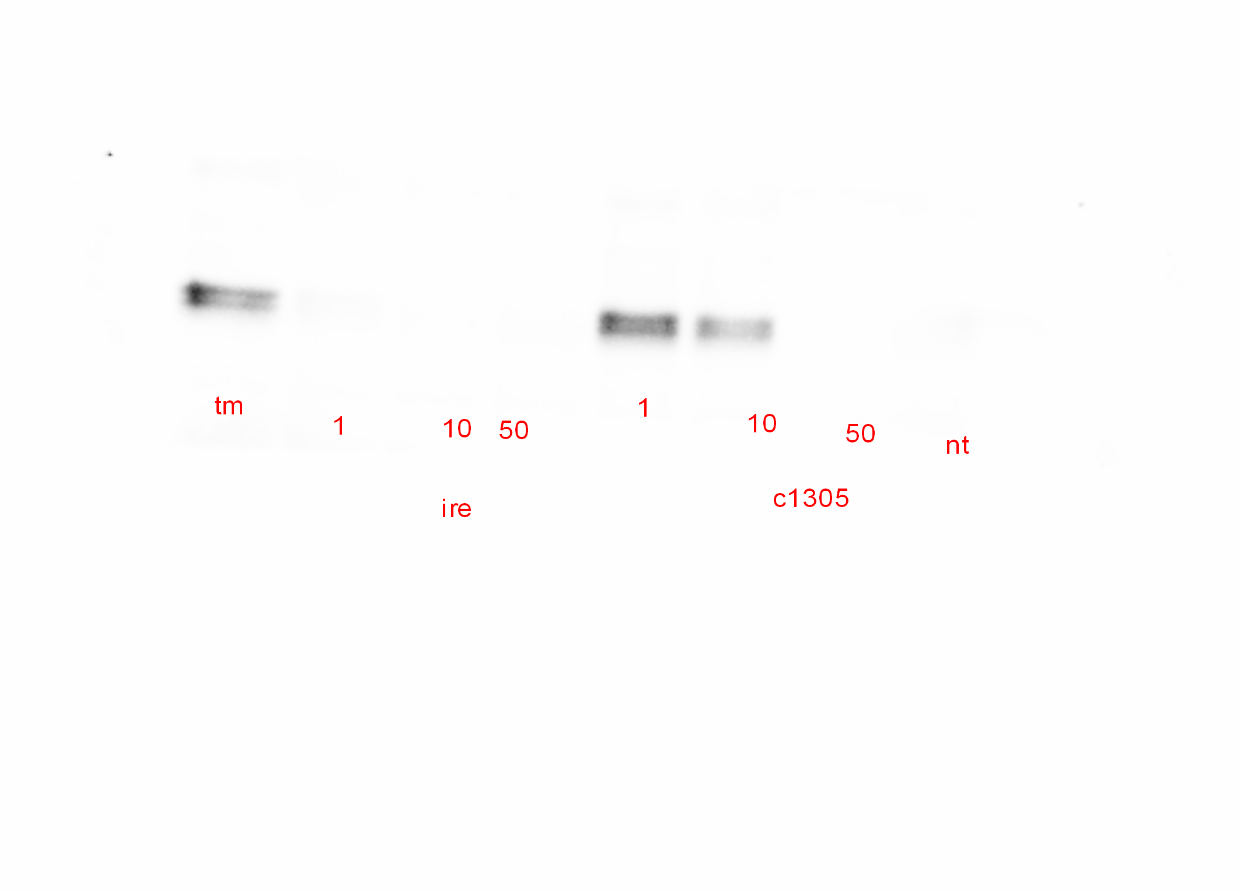


SDS Page


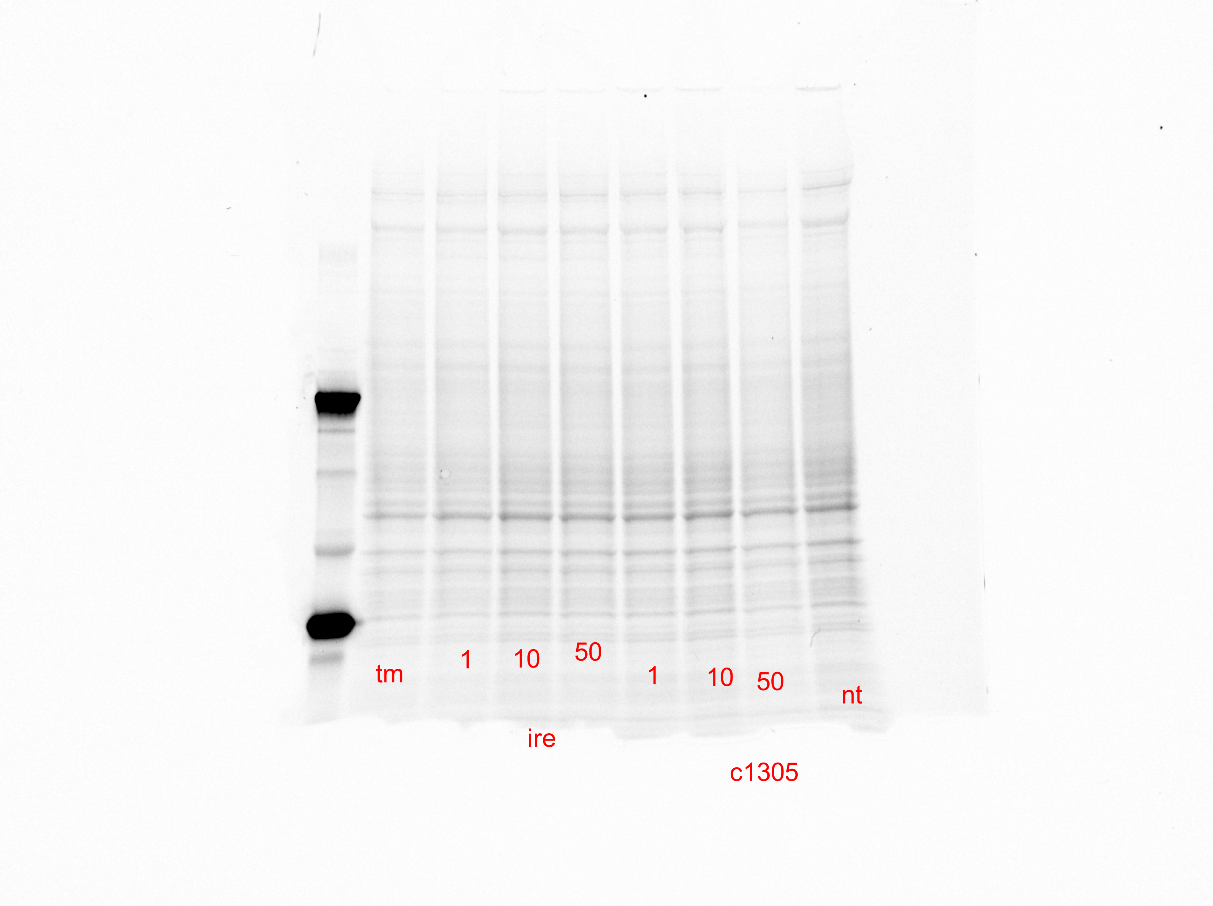

Supplement: Supplementary file 1 — Additional file 1: Evaluation of C-1305 cytotoxicity in HeLa cells exposed for 6 hours to different concentrations of C-1305 and ER stressors, and supplementary materials for Figure 4 (three examples of the uncropped Western blots and SDS Page gels used to follow XBP1s changes). [file 11658_2021_255_MOESM1_ESM.docx]
